# [^68^Ga]Ga-DOTATATE PET/CT and PET/MR enhances the detection of pituitary ACTH-secreting adenomas in cushing’s disease

**DOI:** 10.3389/fendo.2026.1722034

**Published:** 2026-01-29

**Authors:** Zizhen Zhang, Si Xu, Xiaochen Li, Yang Liu, Chang Liu, Jinxin Zhou, Yifan Zhang

**Affiliations:** 1 Department of Nuclear Medicine, Ruijin Hospital, Shanghai Jiao Tong University School of Medicine, Shanghai, China; 2 Department of Nuclear Medicine, Fudan University Shanghai Cancer Center, Shanghai, China

**Keywords:** [^68^Ga]Ga-DOTATATE, cushing’s disease, molecular Imaging, PET, pituitary adenoma

## Abstract

**Introduction:**

This study aimed to evaluate the diagnostic performance of [^68^Ga]Ga-DOTATATE PET/CT or PET/MR in comparison to dynamic MR (dMR) in locating ACTH-secreting pituitary adenomas in Cushing's disease (CD). The utility of PET and MR semi-quantitative parameters was additionally explored as non-invasive biomarkers for tumor characterization.

**Methods:**

Aretrospective analysis was conducted in 14 patients with CD and 16 controls with ectopic ACTH syndrome. Surgical pathology and post-operative follow-up served as reference standard. All patients underwent [^68^Ga]Ga-DOTATATE PET/CT or PET/MR, dMR; some also underwent [^18^F]F-FDG PET. Imaging interpretation included visual and semi-quantitative (SUVmax and ADCmin) assessment.

**Results:**

[^68^Ga]Ga-DOTATATE PET demonstrated a 100% sensitivity indetecting ACTH-secreting pituitary adenomas, superior to dMR with a sensitivity of 85.7%. [^18^F]F-FDG PET showed a sensitivity of 80%. Combining [^68^Ga]Ga-DOTATATE PET with functional tests (high dose dexamethasone-suppression test and/or inferior petrosal sinus sampling) achieved specificity up to 100%, whereas the specificity of PET alone was only 50% due to pituitary structural abnormalities. Semi-quantitative analysis confirmed significantly reduced [^68^Ga]Ga-DOTATATE SUVmax and ADCmin in adenomas. Diagnostic efficacy ranked as follows: G-SUVmax from PET/MR was optimal (AUC1.0), followed by ADCmin (AUC 0.96) and G-SUVmax from PET/CT (AUC 0.87).

**Discussion:**

G-SUVmax and ADCmin can be used as reliable diagnostic predictors. [^68^Ga]Ga-DOTATATE PET/MR may facilitate the preoperative localization of CD.

## Introduction

Cushing’s disease (CD), which is caused by adrenocorticotropic hormone secreting pituitary adenomas, offers significant diagnostic challenges in clinical practice. Biochemical tests are widely used but exhibit limited accuracy in differentiating CD from ectopic Adrenocorticotropic Hormone syndrome (EAS), for example, the high-dose dexamethasone suppression test (DST; high dose DST, HDDST) has approximately 78%–81% sensitivity and 67%–81% specificity in differentiating a pituitary from an ectopic source (1). Although bilateral inferior petrosal sinus sampling (BIPSS) remains the gold standard, it is invasive, operator-dependent, and subject to anatomical variations. False-negative rates range from 3% to 19%, particularly in cyclic or mild CD, though the rate is low during active disease (1–3). These limitations highlight the need for precise imaging localization.

Complete surgical resection is the treatment of choice in patients with confirmed CS, highlighting the crucial role of disease localization (4, 5). Unfortunately, distinguishing CD from EAS remains a challenge in clinical practice of Adrenocorticotropic Hormone(ACTH)-dependent CS (6). Non-invasive approaches such as HDDST and 1-desamino-8-D-arginine vasopressin test (DDAVP) have currently been widely used for differential diagnosis, while these tests sometimes have false or discount results (5). Therefore, imaging localization is also a necessary part of preoperative evaluation.

Pituitary dynamic contrast-enhanced MR (dMR) is currently recommended as the first-line imaging tool by the Consensus on the Management of Cushing’s Syndrome (7). However, given that the majority of pituitary ACTH-secreting tumors are microadenoma, the sensitivity of dMR remains suboptimal, with only 50%–70% CD patients showing a definite or probable abnormal MR finding (1, 7, 8). Even if a pituitary adenoma is identified by dMR, it could be non-functional pituitary tumor, which is not the source of abnormal ACTH production, and often needs supplementary invasive BIPSS (9). Therefore, it is necessary to further explore the role of non-invasive functional imaging modality in precise tumor localization.

Molecular imaging using somatostatin receptor (SSTR)-targeted tracers, such as [^68^Ga]Ga-DOTATATE, offers a promising paradigm shift. Although normal pituitary tissue exhibits physiological SSTR2-mediated [^68^Ga]Ga-DOTATATE uptake, ACTH-secreting adenomas frequently demonstrate lower SSTR expression levels compared to adjacent pituitary parenchyma (10, 11). Conversely, [^18^F]F-FDG positron emission tomography (PET) capitalizes the hypermetabolic nature of ACTH-secreting adenomas, showing increased tracer uptake relative to normal glandular tissue (12–17). The complementary roles of dual radiotracers, highlighting receptor expression and metabolic activity, may synergistically improve diagnostic accuracy when integrated into hybrid PET/MR systems (18, 19).

The advent of simultaneous PET/MR imaging provides unprecedented opportunities for CD diagnosis. By combining the superior soft-tissue contrast of MR (including diffusion-weighted imaging (DWI) and dynamic sequences) with the molecular specificity of PET, this modality allows co-registered assessment of tumor morphology, cellularity, and metabolic/receptor profiles (20, 21).

This study aimed to evaluate the diagnostic performance of [^68^Ga]Ga-DOTATATE PET/CT or PET/MR in comparison to dMR in locating ACTH-secreting pituitary adenomas in CD. The utility of PET and MR semi-quantitative parameters was further explored as biomarkers for tumor characterization.

## Materials and methods

### Patients

**Figure 1:**
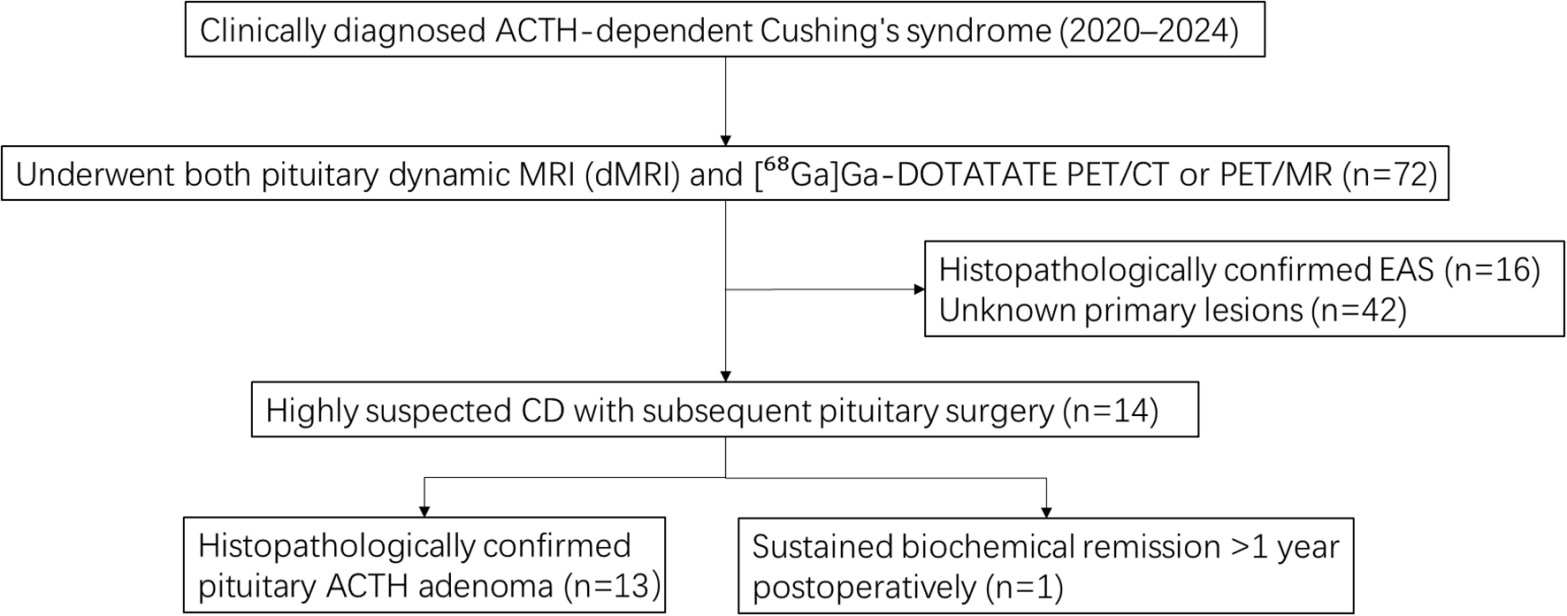
Flowchart.

This retrospective analysis was approved by the Institutional Ethics Committee (No. 2024-300), with a waiver of informed consent. From 72 patients with biochemically confirmed ACTH-dependent Cushing’s syndrome (2020–2024), we included those who fulfilled the following criteria (**Figure 1**): (1) ACTH-dependent hypercortisolism; (2) completion of pituitary dynamic MR (dMR) and [^68^Ga]Ga-DOTATATE PET/CT or PET/MR; and (3) histopathologically confirmed ACTH-secreting pituitary adenoma or sustained biochemical remission after surgery. The final cohort consisted of 14 Cushing’s disease (CD) and 16 ectopic ACTH syndrome (EAS) patients. All CD patients underwent transsphenoidal resection (12 *de novo*, 2 recurrent). [^18^F]F-FDG PET/CT or PET/MR was performed in 5 CD patients and 12 EAS patients based on clinical need.

All patients completed a standardized diagnostic workflow. Biochemical tests—including the high-dose dexamethasone suppression test (HDDST)—and bilateral inferior petrosal sinus sampling (BIPSS), when indicated, were performed prior to [^68^Ga]Ga-DOTATATE PET. These functional assessments aimed to establish the pituitary origin of ACTH excess, whereas imaging was used for lesion localization.

BIPSS was not performed in eight patients for the following reasons: one microadenoma case with a positive DDAVP test; two macroadenomas with mass effect proceeding directly to surgery; and five EAS patients with extraneous primary tumors confirmed on imaging.

### Imaging

#### dMR

Dynamic pituitary MR was obtained in all patients using superconducting magnet 3.0-T scanners (model not fixed). Before gadolinium injection (0.01 mmol/kg gadopentetate dimeglumine, Magnevist; Berlex Laboratories, Montville, NJ, USA), T1-weighted SE and T2-weighted turbo SE images were acquired, followed by coronal dynamic acquisition (T1-weighted turbo SE) in the coronal plane with the following parameters: TR/TE, 400/20 ms; 288×192 matrix; two excitations; 18×18 cm field of view (FOV); 3 mm slice thickness with a 0.3 mm intersection gap. Simultaneously with gadolinium injection, coronal and sagittal T1-weighted SE images were obtained and continued for 2 minutes after the injection. MR response was defined as ‘negative’ if no pituitary adenoma was visible, ‘positive for microadenoma’ if the diameter of pituitary adenoma was <10 mm, or ‘positive for macroadenoma’ if the tumor diameter was ≥10 mm.

#### PET preparation

[^68^Ga]Ga-DOTATATE PET/MR(or PET/CT) and [^18^F]F-FDG PET/MR(or PET/CT) were completed on two different days, and the intervals between the two examinations were within 2 weeks. Before [^18^F]F-FDG examination, patients were required to fast for at least 6 hours and the level of fasting blood glucose should be less than 11.1 mmol/L. For patients with diabetes, insulin should not be used on the day of [¹⁸F]F-FDG examination. The PET/MR or PET/CT imaging was performed 45–90 minutes after intravenous injection radioactivity of 2MBq/kg (total amount ≤ 200 MBq) [^68^Ga]Ga-DOTATATE or 1–2.5 MBq/kg [^8^F]F-FDG.

#### PET/MR imaging

Hybrid PET/MR imaging was performed using the Biograph mMR system (Siemens Healthineers). During the 15-minute PET acquisition, brain MR sequences including sagittal 3D T1-weighted Magnetization Prepared Rapid Gradient Echo (Sag 3D T1-MPRAGE), axial T2-weighted imaging (Ax T2WI), axial fluid-attenuated inversion recovery (Ax FLAIR), axial diffusion-weighted imaging (Ax DWI), coronal T1-weighted imaging (Cor T1WI), coronal T2-weighted imaging (Cor T2WI), and coronal T2-weighted imaging with fat suppression (Cor T2WI-FS) were acquired. Since all patients had undergone standard dMR prior to the examination, no additional contrast-enhanced sequences were performed during the PET/MR session, with detailed MR parameters provided in **Supplementary Table Supplementary 1**. PET images were reconstructed using a point-spread-function (PSF) algorithm (344 × 344 matrix, 4 iterations, 21 subsets, Gaussian filter of 2 mm full width at half maximum). Whole-body PET/MR imaging followed, covering 4–5 bed positions from the skull base to the pelvis (4 minutes per bed position for PET), accompanied by axial T1WI, axial T2WI, and axial DWI sequences.

#### PET/CT imaging

The imaging was performed using a total-body PET/CT μEXPLORER scanner (United Imaging Healthcare, Shanghai, China) with a maximum axial FOV of 194 cm. Whole-body PET/CT scans were acquired over a 5-minute duration. PET image reconstruction utilized an ordered-subsets expectation maximization (OSEM) algorithm incorporating time-of-flight (TOF) and point-spread-function (PSF) modeling, with parameters set as follows: 4 iterations, 20 subsets, a 192 × 192 matrix, FOV sizes of 300 mm for the brain and 600 mm for the body, a slice thickness of 2.886 mm, and a 3-mm Gaussian post-processing filter. For head imaging, the reconstructed CT slices were generated at a thickness of 1.5 mm.

### Imaging analysis

In this study, surgical pathology and postoperative clinical follow-up were considered as the reference standard. Visual and semi-quantitative assessments of [^68^Ga]Ga-DOTATATE PET, [^18^F]F-FDG PET, and dMR images were performed using anonymized DICOM data. All interpretations were conducted blinded to BIPSS and other functional test results to ensure objective evaluation and minimize interpretive bias.

**Figure 2:**
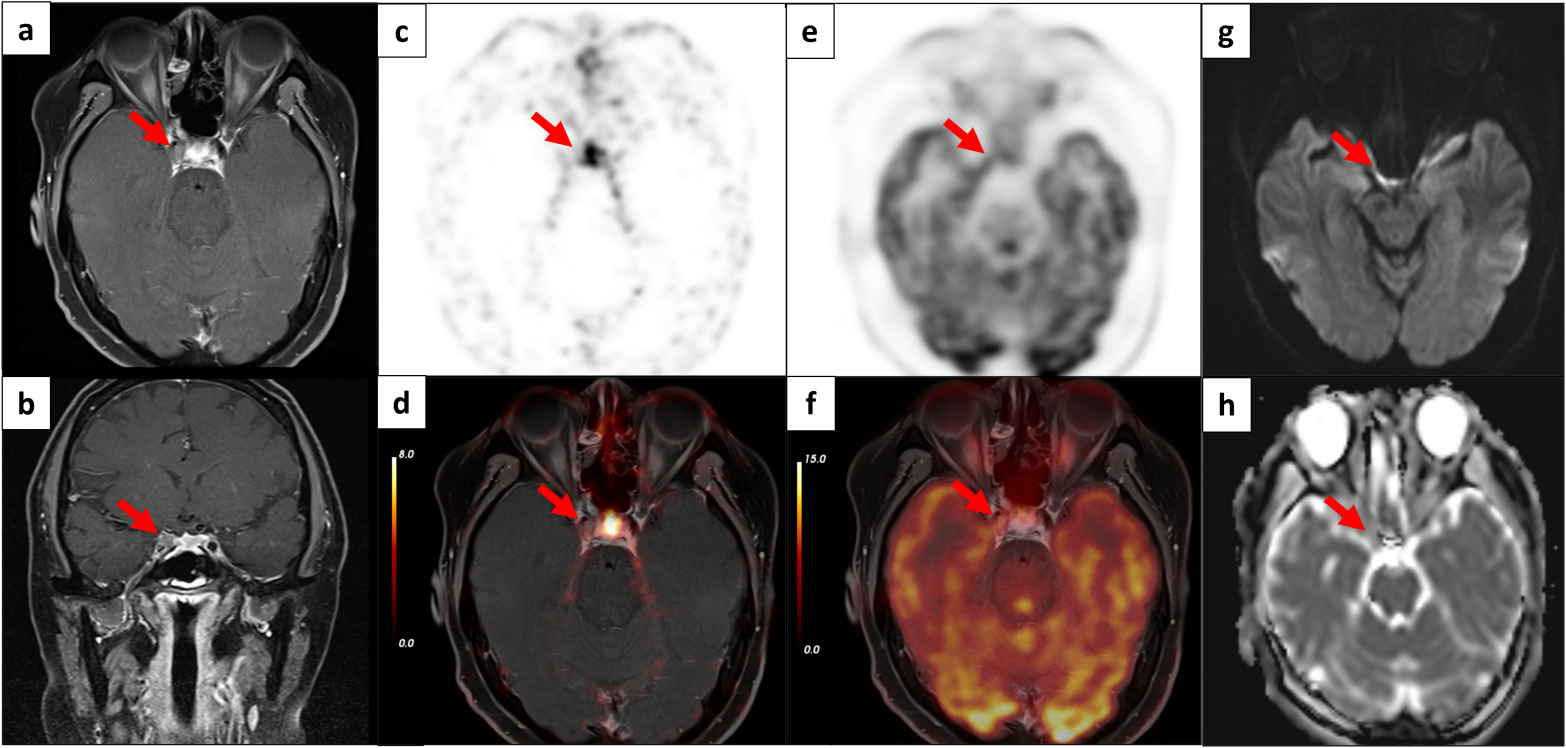
Multimodal PET/MR characterization of a corticotroph adenoma. A 24-year-old woman with CD due to an ACTH-secreting pituitary microadenoma (red arrows). **(a)** Non-contrast axial T1-weighted MR demonstrates a hypointense lesion in the left sella turcica. **(b)** Contrast-enhanced coronal T1-weighted MR reveals non-enhancement of the lesion. **(c, d)** [⁶⁸Ga]Ga-DOTATATE PET and PET/MR fusion images show reduced somatostatin receptor expression in the adenoma (SUVmax 3.60) versus normal pituitary tissue (SUVmax 10.97). **(e, f)** [^18^F]F-FDG PET and PET/MR fusion images demonstrate increased glucose metabolism in the lesion (SUVmax 10.41) compared to contralateral parenchyma (SUVmax 7.79). **(g)** Diffusion-weighted imaging (DWI) displays restricted diffusion with hyperintensity. **(h)** Corresponding ADC map confirms reduced diffusivity (ADCmin 5.1×10⁻⁴ mm²/s vs 11.0×10⁻⁴ mm²/s in normal tissue).

Abnormal [^68^Ga]Ga-DOTATATE uptake was defined as asymmetrically decreased radiotracer uptake in the sella region based on visual assessment. Abnormal [^18^F]F-FDG uptake was defined as localized increased radiotracer uptake in the sella region based on visual assessment. [^68^Ga]Ga-DOTATATE or [¹⁸F]F-FDG PET images were independently reviewed by two experienced nuclear medicine physicians. Discrepancies were resolved by a third senior nuclear medicine physician with over 15 years of experience through re-evaluation and discussion. dMR images were independently reviewed by radiologist using a similar approach. Regions of interest (ROIs) were delineated on the PET images to quantify the maximum standardized uptake value (SUVmax), calculated as the maximum radioactive concentration divided by the injected dose per unit body weight. Specifically, SUVmax measured from [^68^Ga]Ga-DOTATATE PET and [^18^F]F-FDG PET were denoted as G-SUVmax and F-SUVmax, respectively. Corresponding ROIs were also placed on MR images to measure lesion size and minimal apparent diffusion coefficient (ADCmin) values (**Figure 2**). The measurements from those above-mentioned physicians showed no significant difference.

### Statistical analysis

Data were analyzed with SPSS statistical software (version 20; IBM, Armonk, NY, USA). Parameters with a normal distribution were expressed as mean ± standard deviation (SD), while non-normal distribution data were presented as median [range]. Categorical data were described with frequency (percentage). Sensitivity and specificity were derived from 2×2 contingency tables, calculated as TP/(TP+FN) and TN/(TN+FP), respectively. Clopper-Pearson exact CIs were computed for small-sample precision. The differences in semi-quantitative parameters between pituitary adenomas and contralateral normal pituitary tissue were evaluated using paired t-tests for normally distributed data or the Wilcoxon signed-rank test for non-normally distributed data, based on the Shapiro-Wilk test for normality. The optimal cutoff value was determined by receiver-operating-characteristic (ROC) curves. A p-value <0.05 was considered as a statistically significant difference.

## Results

### Patient characteristics

The 14 CD patients had a female predominance (11 females, 78.6%) and a median age of 56 years (range: 22–74). All cases were pathologically confirmed except Case 8, which was clinically diagnosed due to significant biopsy tissue damage; this patient achieved postoperative remission, but MR or pathology could not measure the lesion size. The 16 EAS patients had a female predominance (12 females, 71.4%) and a median age of 53 years (range: 5–69 years) and were all pathologically confirmed. All patients received [^68^Ga]Ga-DOTATATE PET imaging for diagnosis and localization. There were no significant differences in gender, age, or the proportion of PET/CT or PET/MR use between the CD and EAS groups (**Tables 1**, **2**).

**Table 1:** Characteristics of cushing’s disease patients.

CASE	Gender	Age	Diagnostic methods	HDDST	BIPSS	dMR	Diameter (cm)	PET/CT OR PET/MR	[^68^Ga]Ga-DOTATATE PET	[^18^F]F-FDG PET
1	F	22	Pathology	Suppressed	+	+	0.8	PET/MR	+	+
2#	M	40	Pathology	ND	ND	+	4.1	PET/MR	+	+
3	F	63	Pathology	Suppressed	+	+	0.5	PET/MR	+	–
4	F	65	Pathology	Not Suppressed	+	+	2.1	PET/MR	+	+
5	M	74	Pathology	Not Suppressed	ND	+	2.4	PET/MR	+	+
6	F	58	Pathology	Suppressed	ND	+	0.8	PET/CT	+	ND
7*	M	37	Pathology	Not Suppressed	+	+	0.5	PET/CT	+	ND
8	F	57	Clinical	Not Suppressed	+	–	ND	PET/CT	+	ND
9	F	49	Pathology	Suppressed	+	–	0.2	PET/CT	+	ND
10#	F	51	Pathology	ND	+	+	0.6	PET/CT	+	ND
11	F	52	Pathology	Suppressed	–	+	0.7	PET/CT	+	ND
12	F	22	Pathology	Not Suppressed	+	+	0.5	PET/CT	+	ND
13	F	43	Pathology	Not Suppressed	+	+	0.4	PET/CT	+	ND
14*	F	56	Pathology	Suppressed	+	+	0.7	PET/CT	+	ND

ND, not done.

Case 8: Due to significant damage to the pathological tissue, the pathological diagnosis was negative, but clinical evaluation indicated postoperative remission. Both MR and pathological examination revealed no measurable lesions, while PET images were unsuitable for measuring lesion size. Case 9: Tumor size was measured from pathological sections, and the remaining measurements were obtained via dMR.

#Case 2 and Case 10 did not undergo the HDDST due to severe infection and acute thrombotic disease, respectively.

*[^68^Ga]Ga-DOTATATE whole-body PET/CT revealed suspicious peripheral lesions:

Case 7: Focal radiotracer uptake in the pancreatic neck, though contrast-enhanced pancreatic MR showed no abnormalities, ruling out pathology. Case 14: Adrenal adenoma with mild radiotracer uptake, but inconsistent with the clinical diagnosis of ACTH-dependent Cushing's syndrome; ectopic ACTH source excluded.

**Table 2:** Characteristics of ectopic ACTH-secreting syndrome patients.

CASE	Gender	Age	HDDST	BIPSS	dMR	PET/CT OR PET/MR	Pathology
1	F	69	Not Suppressed	ND	Negative	PET/CT	Mediastinal NEC
2	F	33	Not Suppressed	Negative	Rathke's cleft cyst	PET/CT	Mediastinal NET, G1
3	F	55	Not Suppressed	Negative	Partial empty sella	PET/CT	Pulmonary NET, G2
4	F	67	Not Suppressed	Negative	Fullness	PET/CT	Pulmonary NET, G1
5	M	60	Not Suppressed	ND	Negative	PET/MR	Thyroid medullary carcinoma
6	F	46	Not Suppressed	Negative	Empty sella	PET/MR	Mediastinal NEC
7	M	26	Not Suppressed	Negative	Negative	PET/CT	Renal NET, G1
8	F	5	ND	ND	Negative	PET/MR	Thymic NET, G1
9	F	68	Not Suppressed	ND	Negative	PET/MR	Non-small cell lung carcinoma
10	F	33	Not Suppressed	Negative	Negative	PET/CT	Pancreatic NET, G2
11	F	57	ND	ND	Negative	PET/CT	Pulmonary NET, G2
12	F	55	Not Suppressed	Negative	Adenoma	PET/CT	Pancreatic NET, G1(ACTH+)
13	M	53	ND	Negative	Partial empty sella	PET/CT	Thymic NEC
14	M	53	Not Suppressed	Negative	Partial empty sella	PET/CT	Mesenteric NET
15	F	44	Not Suppressed	Positive (IPS/P= 2.63)	Negative	PET/MR	Olfactory neuroblastoma
16	F	26	Not Suppressed	Negative	Rathke's cleft cyst	PET/CT	Thymic NET, G2

ND, not done.

NET, neuroendocrine tumor, well-differentiated.

NEC, neuroendocrine carcinoma.

IPS/P, Ratio of ACTH concentration in the inferior petrosal sinus to peripheral vein.

### Diagnostic performance of HDDST, BIPSS, dMR, and [^18^F]F-FDG PET

Diagnostic performance varied significantly across modalities for Cushing’s disease versus ectopic ACTH syndrome. HDDST demonstrated a limited sensitivity of 50.0% but a perfect specificity of 100.0%. BIPSS achieved a superior sensitivity of 90.9% with specificity of 90.9%.

dMR detected lesions in 12/14 CD with a sensitivity of 85.7% and a specificity of 93.8%, identifying tumors sized 0.4–4.1 cm, while two sub-centimeter lesions (≤2 mm) remained undetectable. In contrast, [^18^F]F-FDG PET alone demonstrated a sensitivity of 80.0% (4/5 patients) for adenomas, a result primarily influenced by lesion size: the detected positive lesions included macroadenomas (≥1 cm) and one 0.8 cm microadenoma, while it failed to visualize a 0.5 cm microadenoma.

### Incremental value of [^68^Ga]Ga-DOTATATE PET

**Figure 3:**
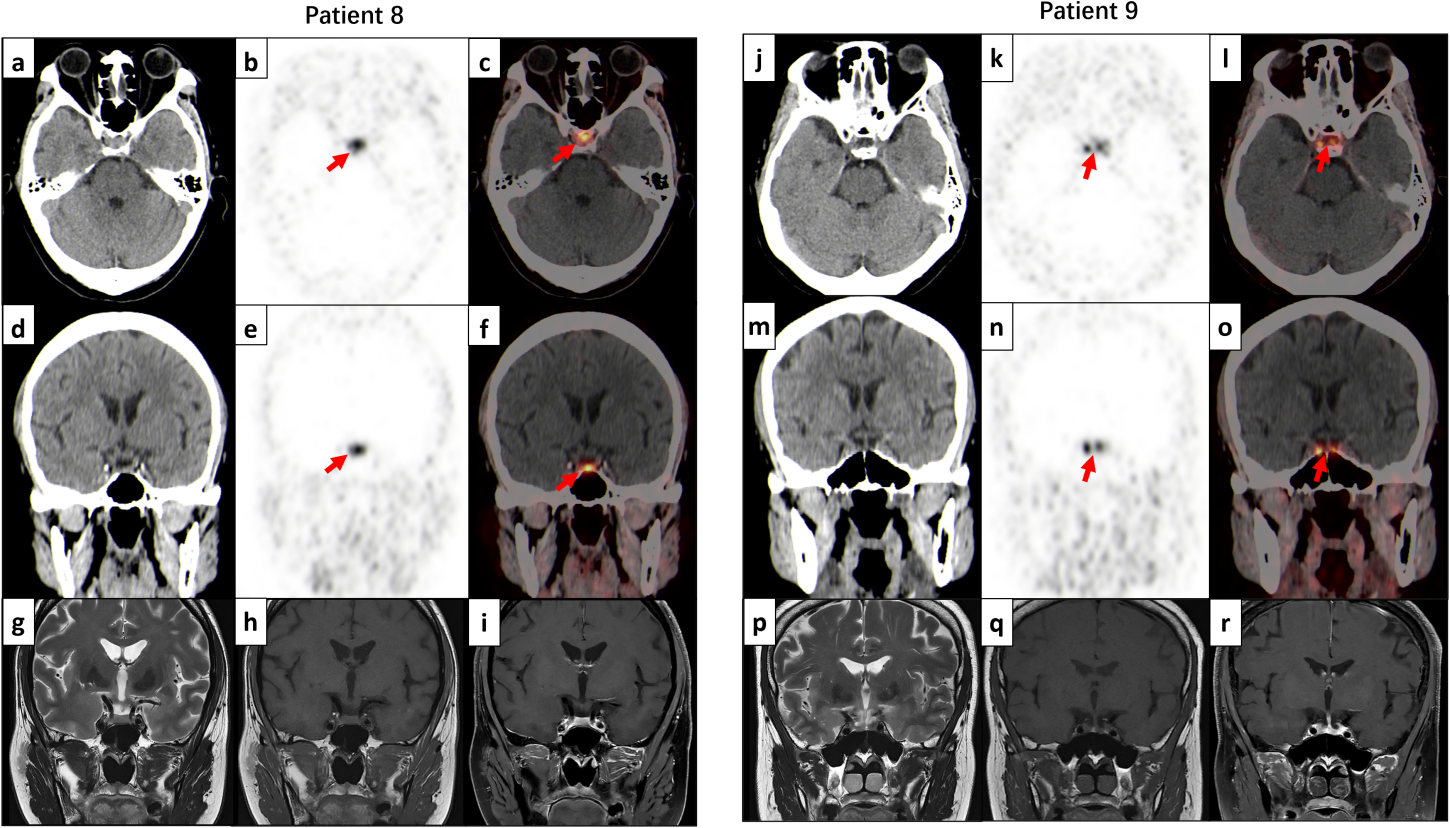
[^68^Ga]Ga-DOTATATE PET/CT detects lesions negative on dMR. Patient 8: HDDST was not suppressed, BIPSS indicated central dominance, but dMR revealed no apparent lesions. [^68^Ga]Ga-DOTATATE PET/CT findings: **(a)** Axial non-contrast CT: No definite space-occupying lesion observed in the sellar region. **(b)** Axial PET image: A focal area of reduced tracer uptake (lesion SUVmax 5.4, red arrow) is noted in the posterior right aspect of the sellar region, compared to normal pituitary tissue (SUVmax 10.3). **(c)** Axial PET/CT fusion: The hypometabolic region corresponds to the right pituitary gland (red arrow). **(d)** Coronal CT, **(e)** Coronal PET, and **(f)** Coronal PET/CT fusion: Further confirm the findings in panels **(a-c)**. On dMRI, the corresponding region shows no abnormal signal on coronal T2-FSE **(g)**, T1-FSE **(h)**, or contrast-enhanced T1-FSE **(i)** sequences. Patient 9: HDDST was suppressed; BIPSS showed central dominance, but dMR revealed no lesions. Pathology confirmed a 0.2 cm microadenoma with positive ACTH expression on immunohistochemistry. [^68^Ga]Ga-DOTATATE PET/CT findings: **(j)** Axial non-contrast CT: Normal pituitary morphology. **(k)** Axial PET image: A photopenic area (lesion SUVmax 4.3, red arrow) is observed in the central sellar region, compared to normal pituitary uptake (SUVmax 10.1). **(l)** Axial PET/CT fusion: Anatomic-functional mismatch localizes to the mid-right pituitary (red arrow). **(m)** Coronal CT, **(n)** Coronal PET, and **(o)** Coronal PET/CT fusion: Correlate with the findings in panels **(j-l)**. On dMRI, the corresponding region shows no abnormal signal on coronal T2-FSE **(p)**, T1-FSE **(q)**, or contrast-enhanced T1-FSE **(r)** sequences.

All 14 patients underwent [⁶⁸Ga]Ga-DOTATATE PET imaging (5 PET/MR, 9 PET/CT), with reduced tracer uptake regions concordant with pathological findings (100% sensitivity), including the 2 dMR-negative cases (**Figure 3**). This confirmed the superior sensitivity of [^68^Ga]Ga-DOTATATE PET over standalone dMR, particularly for MR-indeterminate or negative CD.

Analysis of 16 EAS controls revealed pituitary decreased tracer uptake in 7 cases (3 partial empty sella, 2 Rathke’s cysts, 1 nonfunctional adenoma, 1 indeterminate dMR finding), yielding 100% sensitivity but only 56.3% specificity for [^68^Ga]Ga-DOTATATE PET. However, combining PET with functional tests (HDDST or BIPSS positivity) improved specificity to 100%, emphasizing the diagnostic utility of combined biochemical ad imaging modality in CD.

### Diagnostic value of semi-quantitative parameters in [⁶⁸Ga]Ga-DOTATATE PET and dMR

Semi-quantitative parameters further confirmed the visual diagnostic accuracy. Adenomas exhibited markedly lower [⁶⁸Ga]Ga-DOTATATE uptake compared to contralateral physiologic uptake, as reflected by G-SUVmax values obtained from both PET/MR (3.4 ± 0.2 vs 7.7 ± 1.7, p < 0.01) and PET/CT (4.9 ± 0.8 vs 9.4 ± 1.5, p < 0.05). Additionally, the minimal apparent diffusion coefficient (ADCmin) was significantly reduced in adenomas compared to normal tissue (5.1 ± 0.9 vs 11.0 ± 1.1 ×10⁻⁴ mm²/s, p < 0.01).

**Figure 4:**
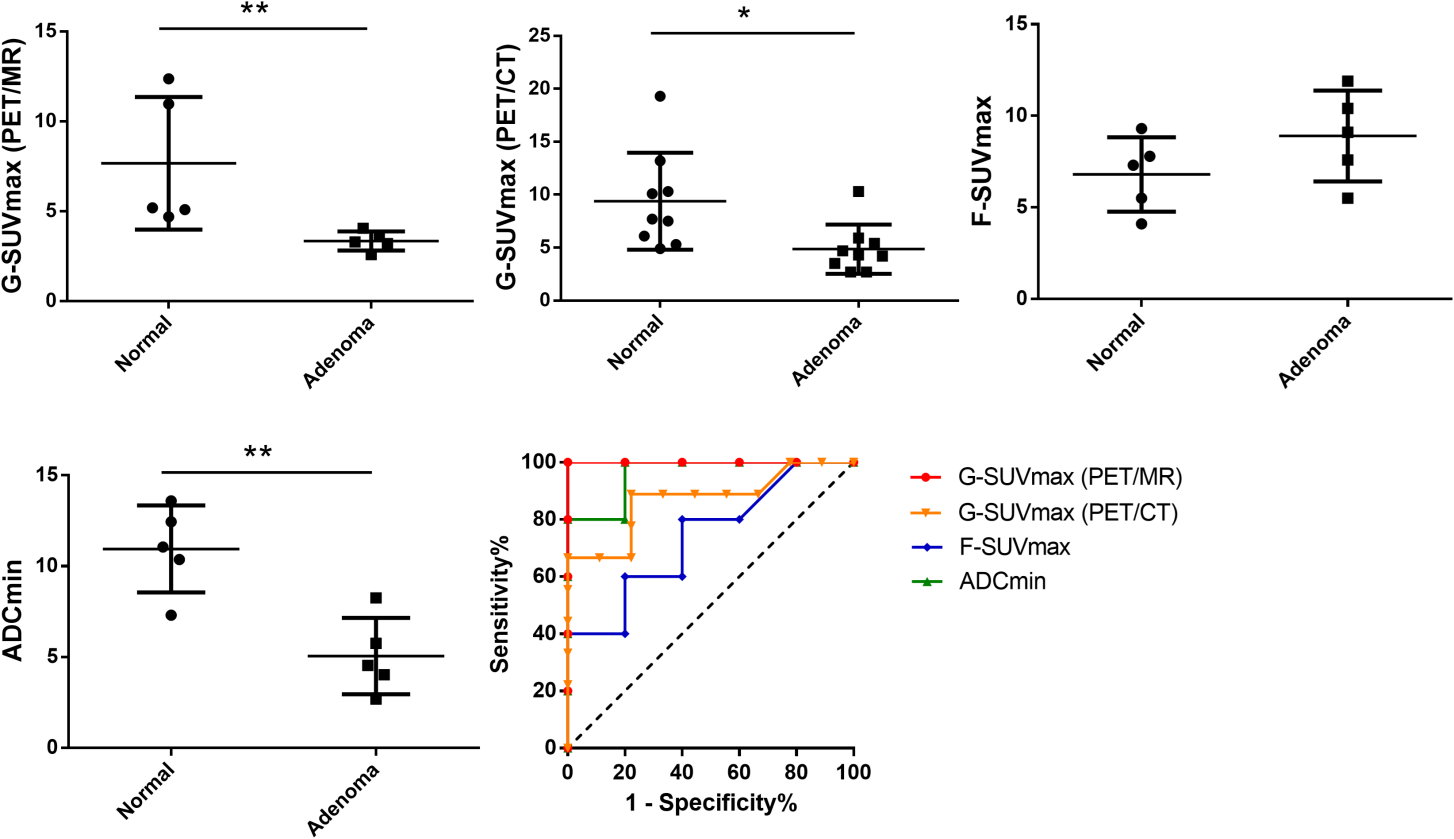
Diagnostic performance of quantitative PET/MR parameters. **(a–b)** SSTR density and G-SUVmax derived from PET/MR were significantly lower in adenomas than in normal pituitary tissue (PET/MR: 3.4 ± 0.2 vs. 7.7 ± 1.7, **p < 0.01; PET/CT: 4.9 ± 0.8 vs. 9.4 ± 1.5, *p < 0.05). **(c)** In contrast, F-SUVmax showed no significant difference between adenomas and normal pituitary tissue (8.9 ± 1.1 vs. 6.8 ± 0.9, p > 0.05). **(d)** ADCmin was markedly reduced in adenomas compared with normal tissue (5.1 ± 0.9 vs. 11.0 ± 1.1 ×10⁻⁴ mm²/s; **p < 0.01). **(e)** ROC analysis revealed superior diagnostic performance for G-SUVmax obtained from PET/MR, which achieved a perfect AUC of 1.0 (N = 5). At a cutoff of <4.38, it distinguished adenomas from normal pituitary tissue with 100% sensitivity and specificity. The performance metrics for the other parameters were: ADCmin (AUC 0.96; cutoff <9.315×10⁻⁴ mm²/s; sensitivity 100%, specificity 80%), G-SUVmax from PET/CT (AUC 0.87; cutoff <6.0; sensitivity 88.9%, specificity 77.8%), and F-SUVmax (AUC 0.74; cutoff >8.45; sensitivity 60%, specificity 80%).

ROC curve analysis was performed to evaluate the diagnostic performance of the parameters (**Figure 4**). Within the constraints of our limited sample size, G-SUVmax derived from PET/MR demonstrated perfect discriminative ability (AUC = 1.0), achieving 100% sensitivity and specificity at an optimal cutoff value of 4.70. The diagnostic performance of both ADCmin (AUC = 0.960, N = 5; cutoff <9.315×10⁻⁴ mm²/s; sensitivity 100%, specificity 80%) and G-SUVmax from PET/CT (AUC = 0.87, N = 9; cutoff <6.0; sensitivity 88.9%, specificity 77.8%) was also excellent. It is important to note that there were no statistically significant differences in the AUC values among these three top-performing parameters (all p > 0.05). Consequently, while G-SUVmax (PET/MR) achieved a perfect score in this cohort, the analysis does not establish the statistical superiority of any one of these three parameters over the others. In contrast, F-SUVmax (AUC = 0.680, N = 5; cutoff >9.10; sensitivity 40%, specificity 80%) demonstrated more moderate diagnostic utility (**Figure 4**).

In addition, we analyzed the potential correlations between disease activity metrics at the time of the PET scan and quantitative PET parameters. Statistical analysis revealed no significant correlations between the levels of serum cortisol (8 AM), plasma ACTH, and urinary free cortisol measured prior to the PET examination and the imaging parameters (G-SUVmax and F-SUVmax from PET, as well as ADCmin values from PET/MR).

## Discussion

It is important to emphasize that while HDDST alone shows perfect specificity in confirming pituitary origin, it provides no anatomical localization. Although pituitary MRI is recommended for all suspected ACTH-dependent Cushing’s syndrome cases, conventional dynamic MRI demonstrates suboptimal sensitivity for microadenomas (6). As previously reported, pituitary ACTH-secreting adenoma can be visualized as decreased uptake on [⁶⁸Ga]Ga-DOTATATE PET (18). In our study, [⁶⁸Ga]Ga-DOTATATE PET achieved 100% sensitivity and successfully identified two dMR-negative cases, highlighting its superior localization capability for microadenomas. However, its limited specificity necessitates correlation with dMR to exclude structural abnormalities like Rathke’s cleft cysts and with biochemical tests to confirm pituitary origin. The [^68^Ga]Ga-DOTATATE PET/MRI provides complementary somatostatin receptor expression and morphological data, enabling comprehensive one-stop preoperative assessment, whereas [^18^F]F-FDG PET remains limited by inadequate metabolic contrast for microadenoma detection.

Beyond traditional imaging, several functional nuclear medicine techniques show promise in diagnosing CD, particularly in challenging cases. ¹¹C-Methionine PET, which highlights regions of increased amino acid metabolism, can aid in localizing pituitary microadenomas, especially when MRI is negative or inconclusive. Co-registration with high-resolution MRI significantly improves detection sensitivity (22). Furthermore, the emerging ⁶⁸Ga-Pentixafor PET, targeting the CXCR4 receptor, has demonstrated potential in distinguishing pituitary from ectopic sources in ACTH-dependent Cushing’s syndrome. Its uptake intensity may correlate with tumor ACTH secretion levels (23, 24). These advanced techniques represent a growing arsenal for precise pre-operative localization.

BIPSS remains the gold standard to identify a pituitary versus ectopic source of excessive ACTH, while such test is invasive and technically demanding with some contraindications. In addition, it has relatively limited value in the lateralization of ACTH-secreting adenomas (6). Combining [^68^Ga]Ga-DOTATATE PET with functional tests can highly improve the specificity of PET imaging, which may reduce the need for invasive BIPSS in some patients. However, it was reported that an incidental pituitary abnormality was seen in up to 40% patients with EAS (6). When there is discordance between [^68^Ga]Ga-DOTATATE PET and HDDST, BIPSS may be inevitable. In that case, whole body [^68^Ga]Ga-DOTATATE PET imaging may also contribute to the identification of ectopic ACTH lesions.

Our study identifies G-SUVmax and ADCmin as promising semi-quantitative parameters for predicting CD lesions. While EAS patients were included for specificity assessment, their pituitary SUVmax values are suboptimal as reference standards due to chronic hypercortisolism potentially altering SSTR expression (25). Our study primarily utilized internal controls (adenoma vs. contralateral normal tissue within the same patient), which better reflects clinical practice and minimizes confounding factors. The significantly lower G-SUVmax in pituitary ACTH-secreting adenomas compared to normal tissue may reflect the reduced expression of SSTR2 in adenomas, as supported by prior molecular analyses (26). The lower ADCmin values in corticotropinomas may similarly indicate increased cellularity and a reduced extracellular space, consistent with observations in other neuroendocrine tumors (27). ROC analysis revealed that G-SUVmax (PET/MR), ADCmin, and G-SUVmax (PET/CT) all achieved high diagnostic accuracy, with no statistically significant differences observed between them in this preliminary dataset. These findings suggest that these parameters may provide valuable adjunctive information for lesion differentiation, warranting further validation in larger cohorts.

When comparing our [⁶⁸Ga]Ga-DOTATATE PET findings with Kim et al.’s [^68^Ga]Ga-DOTATOC study (28), the observed higher sensitivity likely stems primarily from key methodological distinctions: [^68^Ga]Ga-DOTATATE’s superior SSTR2 affinity provides greater contrast for detecting the characteristic SSTR2-downregulated, photopenic adenoma signature (29), and our stringent positivity criterion—focal asymmetrically decreased uptake—directly targets this downregulation, contrasting with criteria encompassing any focal uptake difference. Additionally, cohort differences (notably older, postmenopausal patients herein) may contribute to inter-study variability given potential age/sex hormone influences on SSTR expression, though tracer selection and interpretation criteria represent the dominant factors. The specific impact of demographics warrants dedicated study.

This study has several limitations. First, its retrospective design and the small cohort size may introduce selection bias, although this reflects the low prevalence of Cushing’s disease in clinical practice. Second, variability in imaging protocols (PET/CT vs. PET/MR) and incomplete [^18^F]F-FDG PET data precluded direct comparisons between radiopharmaceuticals and imaging modalities. Third, the exclusion of 42 patients with unknown primary lesions—a population that likely includes occult microadenomas—may have led to overestimation of the sensitivity for both dMR and [^68^Ga]Ga-DOTATATE PET, as these cases lacked a definitive reference standard. Fourth, the ‘asymmetrically decreased uptake’ criterion for PET positivity is inherently susceptible to pituitary physiological heterogeneity, anatomical variations, partial-volume effects, and motion artifacts, necessitating meticulous PET-MRI coregistration and high-resolution MRI as an anatomical reference for reliable interpretation. Of note, while a head-to-head comparison of [^68^Ga]Ga-DOTATATE PET versus dMR (both combined with biochemical testing) was beyond this retrospective study’s scope, such direct strategy comparison holds significant clinical interest. Our findings indicate PET’s superior sensitivity, especially in dMR-inconclusive cases, potentially reducing repeat imaging needs and enhancing surgical precision. Future prospective studies with standardized protocols, larger cohorts, and long-term follow-up of occult cases are warranted to validate these results and better define the diagnostic performance in challenging subpopulations.

## Conclusions

In this retrospective study with a limited sample size, [^68^Ga]Ga-DOTATATE PET demonstrated higher sensitivity than dMR for detecting ACTH-secreting adenomas, particularly in dMR-negative cases, suggesting a complementary role. However, its specificity alone was limited (50%) and reached 100% only when integrated with confirmatory functional tests (e.g., HDDST/BIPSS). The semi-quantitative parameters G-SUVmax and ADCmin show promise as non-invasive biomarkers. These findings support the use of [^68^Ga]Ga-DOTATATE PET as a valuable adjunct to dMR in selected cases, especially when conventional imaging is inconclusive, but underscore the necessity of combining functional testing for definitive localization.

## Data Availability

The original contributions presented in the study are included in the article/**Supplementary Material**. Further inquiries can be directed to the corresponding author/s.
