# Supplementary material for: [68Ga]Ga-DOTATATE PET/CT and PET/MR enhances the detection of pituitary ACTH-secreting adenomas in cushing’s disease
Source: Front Endocrinol (Lausanne). 2026 Jan 29;17:1722034. doi: 10.3389/fendo.2026.1722034 (PMC12893962; doi:10.3389/fendo.2026.1722034)
Supplement: Supplementary file 1 [file Table1.docx]

**Supplement 1. The implementation details of head MR for Cushing Syndrome patients**

| Sequences | 3D T1-weighted Magnetization prepared Rapid Gradient Echo | T2-weighted Fluid-Attenuated Inversion Recovery | Diffusion-weighted Imaging |
| --- | --- | --- | --- |
| Repetition time | 1900 ms | 8500 ms | 5900 ms |
| Echo time | 2.44 ms | 92 ms | 85 ms |
| Slice thickness | 1.0 mm | 2.0 mm | 5.0 mm |
| Acquisition matrix | 256×256 | 256×256 | 146×146 |
| B value | - | - | 1000 |

**Supplement 2. Semiquantitative PET Parameters and Pre-scan Hormone Levels**

|  | Gender | Age | 24h UFC (μg/24h) | 24-hour urine volume (ml) | 8AM-cortisol (μg/dl) | 8AM-ACTH (pg/ml) | Diameter (cm) | PET | Lesion G-SUVmax | Normal Pituitary G-SUVmax | Lesion F-SUVmax | Normal Pituitary F-SUVmax | Lesion-ADCmin | Normal Pituitary-ADCmin |
| --- | --- | --- | --- | --- | --- | --- | --- | --- | --- | --- | --- | --- | --- | --- |
| 1 | F | 22 | 3778.88 | 1600 | 101.21 | 126.7 | 0.8 | PET/MR | 3.6 | 10.97 | 10.41 | 7.79 | 2.69 | 7.3 |
| 2 | M | 40 | ND | ND | ND | 900.79 | 4.1 | PET/MR | 2.6 | 5.2 | 11.9 | 9.3 | 4.54 | 10.37 |
| 3 | F | 63 | 555.39 | 2700 | 27.51 | 71.14 | 0.5 | PET/MR | 3.3 | 5.1 | 5.5 | 5.5 | 8.26 | 13.59 |
| 4 | F | 65 | 1429.39 | 1900 | 33.91 | 214.9 | 2.1 | PET/MR | 4.05 | 12.37 | 9.1 | 7.3 | 4.03 | 12.44 |
| 5 | M | 74 | 2578.37 | 1350 | 38.79 | 815.6 | 2.4 | PET/MR | 3.2 | 4.7 | 7.6 | 4.11 | 5.75 | 11.05 |
| 6 | F | 58 | 278.88 | 1200 | 20.04 | 72.75 | 0.8 | PET/CT | 5.9 | 13.2 | NA | NA | NA | NA |
| 7 | M | 37 | 1518.88 | 1600 | 25.64 | 103.66 | 0.5 | PET/CT | 10.3 | 19.3 | NA | NA | NA | NA |
| 8 | F | 57 | ND | ND | 25.87 | 149.5 | NA | PET/CT | 5.4 | 10.3 | NA | NA | NA | NA |
| 9 | F | 49 | 182.52 | 2600 | 22.62 | 49.2 | 0.2 | PET/CT | 4.3 | 10.1 | NA | NA | NA | NA |
| 10 | F | 51 | 992.43 | 1750 | 23.01 | 116.32 | 0.6 | PET/CT | 4.2 | 7.5 | NA | NA | NA | NA |
| 11 | F | 52 | 120.12 | 1200 | 16.9 | 78 | 0.7 | PET/CT | 3.5 | 5.3 | NA | NA | NA | NA |
| 12 | F | 22 | 424.4 | 2000 | 26.81 | 97.6 | 0.5 | PET/CT | 2.7 | 6.1 | NA | NA | NA | NA |
| 13 | F | 43 | 451.49 | 1300 | 21.41 | 115.6 | 0.4 | PET/CT | 2.7 | 4.9 | NA | NA | NA | NA |
| 14 | F | 56 | 248.16 | 1600 | 12.98 | 50.33 | 0.7 | PET/CT | 4.7 | 7.7 | NA | NA | NA | NA |

ND: The measured value was above the upper detection limit and therefore not quantifiable.

NA: Not Applicable
